# *Helicobacter pylori* Infection and Anemia in Taiwanese Adults

**DOI:** 10.1155/2013/390967

**Published:** 2013-11-18

**Authors:** Hsiang-Yao Shih, Fu-Chen Kuo, Sophie S. W. Wang, Yi-Chang Liu, Meng-Chieh Wu, Yang-Pei Chang, Guei-Fen Chiu, Pi-Yu Chang, Deng-Chyang Wu, Ming-Chia Hsieh, Yao-Li Chen

**Affiliations:** ^1^Department of Internal Medicine, Kaohsiung Municipal Hsiao-Kang Hospital, Kaohsiung City 812, Taiwan; ^2^Division of Gastroenterology, Department of Internal Medicine, Kaohsiung Medical University Hospital, Kaohsiung City 807, Taiwan; ^3^School of Medicine, College of Medicine, I-Shou University and E-Da Hospital, Kaohsiung City 824, Taiwan; ^4^Graduate Institute of Public Health, Kaohsiung Medical University, Kaohsiung City 807, Taiwan; ^5^Division of Hematology and Oncology, Department of Internal Medicine, Kaohsiung Medical University Hospital, Kaohsiung City 807, Taiwan; ^6^Department of Neurology, Kaohsiung Medical University Hospital, Kaohsiung City 807, Taiwan; ^7^Department of Information, Kaohsiung Municipal Ta-Tung Hospital, Kaohsiung City 801, Taiwan; ^8^Administration Center, Kaohsiung Medical University Hospital, Kaohsiung City 807, Taiwan; ^9^Cancer Center, Kaohsiung Medical University Hospital, Kaohsiung City 807, Taiwan; ^10^Division of Endocrinology and Metabolism, Department of Internal Medicine, Changhua Christian Hospital, Changhua County 500, Taiwan; ^11^Graduate Institute of Integrated Medicine, China Medical University, Taichung City 404, Taiwan; ^12^Department of General Surgery, Changhua Christian Hospital, Changhua County 500, Taiwan; ^13^School of Medicine, Kaohsiung Medical University, Kaohsiung City 807, Taiwan

## Abstract

*Background*. Chronic *Helicobacter pylori* infection and iron-deficiency anemia (IDA) are common in adults. Although the most common causes of IDA usually arise from the gastrointestinal tract, the association between chronic *Helicobacter pylori* infection and anemia remains unclear. *Aim*. To evaluate the association of chronic *Helicobacter pylori* infection and IDA. *Materials and Methods*. We enrolled 882 patients from January 2010 to April 2013. The status of *Helicobacter pylori* (H.p) infection was confirmed and blood samples from the same participants were taken on the same day to check the level of hemoglobin, serum iron, ferritin, and total iron-binding capacity (TIBC). *Results*. No significant difference was noted from the demographic data. The average level of hemoglobin (Hb) was not different between negative and positive groups, pos 13.57 g/dL versus neg 13.65 g/dL (*P* = 0.699). Although the levels of serum IDA related parameters were expected in positive group (lower serum iron and ferritin and higher TIBC) these differences did not reach statistical significance (*P* = 0.824 for iron, *P* = 0.360 for ferritin, and *P* = 0.252 for TIBC). *Conclusion*. Chronic *Helicobacter pylori* infection is not attributed to IDA. The levels of hemoglobin, serum iron and ferritin, and TIBC remain unaffected after chronic H.p infection. Large-scale clinical studies are needed to prove the association.

## 1. Introduction

Chronic *Helicobacter pylori* (H.p) infection is responsible for many alimentary tract disorders, including gastroduodenal ulcer, atrophic gastritis, intestinal metaplasia, gastric mucosa-associated lymphoid tissue lymphoma (MALT lymphoma), and gastric adenocarcinoma [1]. Furthermore, it has been implicated in some extragastric diseases, such as unexplained iron-deficiency anemia (IDA), idiopathic thrombocytopenic purpura (ITP), and vitamin B12 deficiency [2, 3].

Iron-deficiency anemia (IDA) is the most common cause of anemia in the world and 500 to 600 million people are affected. IDA is also the most common nutritional deficiency in undeveloped and developed worlds and possibly the most common organic disorder in clinical practice [4, 5]. Anemia is a common manifestation of various etiologies, for example, iron-deficiency, vitamin B12 deficiency, folic acid deficiency, chronic illness, gastrointestinal bleeding, and so forth. Generally, it is attributed to three different pathogenic processes: (1) marrow production defects (hypoproliferation); (2) red cell maturation defects (ineffective erythropoiesis); and (3) decreased red cell survival (blood loss/hemolysis). Consequently, chronic H.p infection is likely to result in anemia. For example, chronic inflammation due to chronic H.p infection is one of the causes which have been related marrow production defects (hypoproliferation) [6]. Iron-deficiency anemia (IDA) is a common cause which occurs in 2–5% of adult males and postmenopausal females in developed countries [7, 8], and most of them resulted from gastrointestinal lesions. Chronic H.p infection usually causes a wide range of gastrointestinal mucosal lesions, such as chronic erosive gastritis, and is very likely to contribute to IDA in affected patients. In addition, chronic H.p infection frequently results in atrophic gastritis, which leads to hypo- or achlorhydria, which is the underlying cause for decreased iron absorption and increased iron uptake and utilization by the bacteria [4].

Despite all the assumptions, no strong evidence from clinical studies is available. Therefore, we hypothesize that chronic H.p infection is related to anemia and have conducted this prospective study to clarify the association between H.p infection and anemia.

## 2. Patients and Methods

### 2.1. Study Design and Patients

Initially, one thousand two hundred and eighteen patients (489 men and 729 women) were enrolled from gastroenterology clinics of three different hospitals, including Kaohsiung Medical University Hospital, Kaohsiung Municipal Hsiao-Kang Hospital, and Kaohsiung Municipal Tatung Hospital, from January 2010 to April 2013. All patients received the esophagogastroduodenoscopy (EGD) examination, and endoscopic biopsy from gastric mucosa was undertaken for confirmation of H.p infection. Exclusion criteria for H.p infection included use of antibiotics, bismuth, or proton pump inhibitor (PPI) within 4 weeks, previous gastric surgery, and history of eradication of H.p. Besides blood was drawn from all of them for hemoglobin, serum iron, serum total iron-binding capacity (TIBC) and serum ferritin checks on the same day. Exclusion criteria for anemia included past history of anemia with known etiology other than H.p, known hematologic disorder causing anemia, evident gastrointestinal bleeding within one month, and evident blood loss within one month. After exclusion, we enrolled 882 cases for further analysis. As for the evaluation of chronic H.p infection and iron-deficiency anemia, we just enrolled 770 cases due to the patients' unwillingness to be checked for serum iron level.

### 2.2. Diagnosis of *H. pylori* Infection

We used culture, histology, rapid urease test, and 13C-urea breath test (UBT) in this study. The Columbia blood agar plate is made use of for culture for endoscopic biopsy specimens. The culture demonstrated positive if one or more colonies showed Gram-negative, oxidase (+), catalase (+), urease (+), or spiral or curved rods in morphology. We also evaluated the presence of *H. pylori* in the histology of gastric biopsy specimens by experienced pathologists. If the color of rapid urease test (sensitivity 93–97%, specificity 98%) [9], CLO test (Delta West Bentley, WA, Australia), turned pink or red at room temperature 6 hours after the EGD examination, it was interpreted as positive. The 13C-urea breath test used in this study was from the Institute of Nuclear Energy Research, Taiwan. The definition of positive *H. pylori* infection was that either culture was positive or at least two positive results of rapid urease test, histology, or UBT [10, 11].

### 2.3. Definition of Anemia

Anemia was defined as serum hemoglobin (Hb) <14 g/dL in males and <12 g/dL in females. The definition of iron-deficiency anemia (IDA) was serum iron <30 *μ*g/dL and total iron-binding capacity (TIBC) >400 *μ*g/dL [6]. We also analyzed the association between chronic H.p infection and iron-deficient erythropoiesis as definition of serum iron <50 *μ*g/dL and total iron-binding capacity (TIBC) >380 *μ*g/dL [6]. Normal ranges of serum iron, TIBC, and ferritin are 45–182 *μ*g/dL (male)/28–170 *μ*g/dL (female), 257–421 *μ*g/dL (male)/254–450 *μ*g/dL (female), and 24–336 ng/mL (male)/11–307 ng/mL (female), respectively.

### 2.4. Statistical Analysis

The demographic characteristics and average serum iron, TIBC, and ferritin levels were analyzed by Student's *t*-test. The relationships between H.p infection and anemia and IDA and iron-deficient erythropoiesis were analyzed by Chi-square test. Statistical significance was considered as *P* < 0.05.

## 3. Results

### 3.1. Demographic Characteristics

A total of 882 patients were enrolled into the study. The average ages of negative and positive H.p infection groups were 57.6 ± 12.7 and 57.5 ± 12.4 years old, respectively, ranging from 21 to 88 years old (Table 1). No significant difference of the demographic characteristics, including age, sex, cigarette smoking, hypertension and cerebrovascular disease, was demonstrated between negative and positive H.p infection groups.

### 3.2. H.p Infection and Anemia

The average level of serum hemoglobin (Hb) was 13.65 g/dL in negative group and 13.57 g/dL in positive group. We analyzed the relationship between H.p infection and mean Hb and anemia (defined as serum Hb < 14 g/dL in males and <12 g/dL in females), and no significant difference was noted, even though the level of Hb was lower in the positive group (*P* = 0.699) (Table 2).

### 3.3. H.p Infection and Iron, Total Iron-Binding Capacity (TIBC), Ferritin, Iron-Deficiency Anemia (IDA) and Iron-Deficient Erythropoiesis

We also evaluated the serum iron, total iron-binding capacity (TIBC), and ferritin of the patients after the enrollment. In the positive H.p infection group, several lines of evidence of IDA, such as lower serum iron, lower serum ferritin, and higher TIBC, were demonstrated but did not reach statistical significance (Table 3). Furthermore, we performed subgroup analyses as serum iron <30 *μ*g/dL, TIBC >400 *μ*g/dL, and ferritin <15 ng/mL, according to the standard definition of IDA. Although more patients in the positive groups had higher TIBC and lower ferritin, there was no significant difference after subgroup analysis (Table 3).

In this study, we enrolled four cases of IDA in the negative H.p infection group but no case in the positive group. With respect to iron-deficient erythropoiesis, twenty and five cases were collected into the negative and positive H.p infection groups individually. Again, no statistical significance was noted between the negative and positive groups (Table 4).

## 4. Discussion

In this study, among the 882 patients, we showed no significant association between chronic *Helicobacter pylori* (H.p) infection and anemia. Although we observed lower levels of hemoglobin in the positive H.p infection group, there was no statistical significance. Also, we showed no significant association between chronic H.p infection and iron-deficiency anemia (IDA), despite lower levels serum iron and ferritin and higher levels of TIBC levels in the positive H.p infected group. There was no significant association between chronic H.p infection and iron-deficient erythropoiesis, either.

Presumably, chronic H.p infection is very likely to cause anemia, given its nature of chronic infection in adults and predisposition to gastrointestinal mucosal lesions, both of which have been attributed as the common causes of anemia [12]. Our data have agreed with previous reports on no significant association between chronic H.p infection and anemia [13–16]. Some studies have reported decreased iron store in positive H.p infection populations [17–20]. We also noted all the features of iron storage, such as lower levels of serum iron and ferritin and higher levels of total iron-binding capacity (TIBC). However, we failed to show the statistical significance.

Serum ferritin is the major storage protein for iron and the most powerful parameter for diagnosis of iron-deficiency anemia without inflammation [7]. Concomitant inflammation can greatly affect the level of serum ferritin. The level of ferritin for iron-deficiency varies from 12–15 ng/mL without concomitant inflammation to more than 50 ng/mL with concomitant inflammation [21, 22]. The levels of serum ferritin are liable to change under many conditions, including chronic inflammation, hyperthyroidism, malignancy (leukemia, Hodgkin's disease), and even type 2 diabetes mellitus. Although many studies reported a lower level of serum ferritin in patients with chronic H.p infection, our data failed to show the significant association between the lower level of serum ferritin and chronic H.p infection in our study.

In this study, there are several limits. The relatively small sample size is not able to portray the relatively subtle difference among all the parameters. We did not exclude most of the concomitant conditions which would confound the parameters, such as the level of ferritin. Therefore, more detailed studies are needed and may help to delineate the changes in levels of hemoglobin, iron, and ferritin after chronic H.p infection and the possible reversal after H.p eradication.

## Figures and Tables

**Table 1:** Demographic characteristics between negative and positive H.p infection groups.

	H.p (−) (*n* = 762)	H.p (+) (*n* = 120)	*P* value
Age(years)			
Mean ± SD	57.6 ± 12.7	57.5 ± 12.4	0.936
Sex			0.939
Male	302 (39.6%)	48 (40%)	
Female	460 (60.4%)	72 (60%)	
Smoking	63 (8.3%)	12 (10%)	0.528
Hypertension	184 (24.1%)	30 (25%)	0.84
Cerebrovascular disease	65 (8.5%)	8 (6.7%)	0.492

*H.p (−) as negative H.p infection and H.p (+) as positive H.p infection.

**Table 2:** The relationship between H.p infection and hemoglobin (Hb) and anemia.

	H.p (−) (*n* = 762)	H.p (+) (*n* = 120)	*P* value
Mean Hb g/dL	13.65	13.57	0.699
Anemia (−)	599 (78.6%)	99 (82.5%)	0.397
Anemia (+)	163 (21.4%)	21 (17.5%)	

Anemia: male: Hb < 14 g/dL, female: Hb < 12.

**Table 3:** The association between H.p infection and iron, ferritin, and TIBC.

	H.p (−) (*n* = 660)	H.p (+) (*n* = 110)	*P* value
Mean iron (*μ*g/dL)	95.28	94.41	0.824
Mean ferritin (ng/mL)	138.07	127.41	0.360
Mean IBC (*μ*g/dL)	330.98	337.68	0.252
Iron < 30	15 (2.3%)	2 (1.8%)	0.765
Iron 30	645 (97.7%)	108 (98.2%)	
TIBC > 400	63 (9.5%)	14 (12.7%)	0.301
TIBC < 400	597 (90.5%)	96 (87.3%)	
Ferritin < 15	39 (5.9%)	7 (6.4%)	0.849
Ferritin > 15	621 (94.1%)	103 (93.6%)	

**Table 4:** The association between H.p infection and IDA and iron-deficient erythropoiesis.

	H.p (−) (*n* = 660)	H.p (+) (*n* = 110)	*P* value
IDA (+) (iron < 30, TIBC > 400)	4	0	
IDA (−)	656	110	
Iron-deficient erythropoiesis (+) (iron < 50, TIBC > 380)	20	5	0.385
Iron-deficient erythropoiesis (−)	640	105	
